# Assessment of Pulmonary Functions in Parkinson's Disease and Unveiling the Role of Levodopa Therapy: A Cross-Sectional Study

**DOI:** 10.7759/cureus.58662

**Published:** 2024-04-20

**Authors:** Om Mishra, Ashok K Mallik, Santosh Kumar Dash, Pragateshnu Das, Manoranjan Dash

**Affiliations:** 1 Neurology, Kalinga Institute of Medical Sciences, Bhubaneswar, IND; 2 Neurology, SCB (Srirama Chandra Bhanja) Medical College and Hospital, Cuttack, IND; 3 Pulmonary Medicine, SCB (Srirama Chandra Bhanja) Medical College and Hospital, Cuttack, IND

**Keywords:** modified hoehn and yahr scale, parkinson's uk brain bank criteria, pulmonary function test (pft), forced vital capacity (fvc), parkinson’s disease, forced expiratory volume in 1 second (fev1), peak expiratory flow rate (pefr)

## Abstract

Introduction: This investigation aimed to thoroughly characterize the range of pulmonary function abnormalities present in individuals with Parkinson's disease (PD) and to evaluate the effects of levodopa therapy on these respiratory dysfunctions.

Methods: Ninety-five PD patients diagnosed via the UK Parkinson's Disease Society Brain Bank Diagnostic Criteria were recruited, excluding those with a smoking history or unable to perform pulmonary function tests (PFTs). Severity was assessed using the Hoehn and Yahr Scale. Spirometry-measured PFT parameters (forced vital capacity (FVC), forced expiratory volume in the first second (FEV1), and peak expiratory flow rate (PEFR)) were compared against matched predicted values. The changes in PFT parameters post-levodopa challenge were assessed.

Results: Most of the PD patients were aged between 51-60 years, with a mean age of 55.89 ± 8.37 years. Of these, 65.3% were male. A significant proportion of the cohort exhibited restrictive pulmonary patterns (73.7%), while a smaller fraction displayed obstructive (7.4%) or normal (18.9%) pulmonary function patterns. Notably, levodopa treatment correlated with marked improvements in all measured PFT parameters, especially evident in the enhancements from the "off" medication stage to the "on" stage for FVC and FEV1 (P=0.0001). A weak positive correlation between the severity of respiratory restriction and the duration of PD (r = 0.139, P = 0.021) was found, suggesting that PD's progression exerts an increasingly adverse effect on respiratory function over time.

Conclusion: The findings of this study illustrate that restrictive pulmonary abnormalities are more prevalent than obstructive patterns in PD patients and that these patients respond favorably to levodopa therapy.

## Introduction

Parkinson's disease (PD) is a progressive neurodegenerative disorder that disrupts motor functions, causing tremors, rigidity, bradykinesia, and postural instability [1]. Beyond these characteristic symptoms, respiratory dysfunction is a significant and often underrecognized aspect of PD that can greatly impact patient health and well-being [2]. While early research suggested obstructive respiratory patterns due to parasympathetic imbalances [3], more recent studies highlight restrictive pulmonary dysfunction as a prevalent issue in PD patients [4]. Levodopa remains the cornerstone of PD treatment, effectively managing motor symptoms [5]. However, its long-term use can lead to motor fluctuations and involuntary movements (dyskinesia), posing a therapeutic challenge [5]. The impact of levodopa on respiratory function is a complex issue. Some studies suggest little to no improvement, or even a worsening of respiratory symptoms, with levodopa [6,7]. Others, however, indicate potential benefits [8]. These conflicting findings emphasize our study's need for a comprehensive understanding of the diverse respiratory problems in PD and how they may be influenced by levodopa therapy.

## Materials and methods

This observational study was carefully designed to investigate pulmonary function abnormalities in PD patients and to explore the effect of levodopa therapy on these parameters. The study was conducted at the Neurology Department of SCB Medical College, Cuttack, Odisha, India, from March 2021 to March 2023. It enrolled 95 PD patients following rigorous criteria to ensure a detailed analysis of pulmonary health in this specific patient group. The study was approved by the Institutional Ethics Committee of SCB Medical College & Hospital (approval number: IEC-1079).

Inclusion and exclusion criteria

Participants were selected based on strict inclusion and exclusion guidelines. The diagnosis of all patients conformed to the UK Parkinson's Disease Society Brain Bank Diagnostic Criteria [9]. The study excluded patients with histories of smoking, existing pulmonary or cardiovascular diseases, medication-induced pulmonary dysfunction, those who did not give consent, and individuals unable to perform pulmonary function tests (PFTs).

Pulmonary function assessment

A comprehensive clinical assessment, including a detailed clinical history and physical examination, was conducted for each participant. The severity of their condition was assessed using the modified Hoehn and Yahr scale [10]. Participants also underwent spirometry tests in line with the American Thoracic Society's standards, which involved measurements like a 7-liter volume capacity, a flow rate of 12 liters per second, calibration using a 3-liter syringe, and recording key parameters such as the minimum forced expiratory volume in the first second (FEV1)/forced vital capacity (FVC) ratio, volume-time curve, and flow-volume loop [11]. The primary PFT parameters measured included FVC (the total volume of air exhaled forcefully), FEV1 (the volume of air expelled in the first second of a forceful exhale), peak expiratory flow rate (PEFR) or the highest speed of airflow during an exhale, and the FEV1/FVC ratio, which is indicative of potential airway obstruction. Pulmonary function was classified according to American Thoracic Society (ATS) guidelines into normal function, marked by an FEV1/FVC ratio above 70% and an FVC% predicted at 80% or higher, restrictive lung pattern characterized by an FEV1/FVC ratio above 70% but an FVC% predicted below 80%, and obstructive lung pattern, characterized by an FEV1/FVC ratio below 70%.

Levodopa impact evaluation

To determine the effects of levodopa on pulmonary function, PFTs were carried out in "on" and "off" medication states. The "off" state followed a 12-hour withdrawal from levodopa, while the "on" state was assessed 60 minutes after levodopa administration. This methodology provided insight into levodopa's immediate influence on pulmonary function.

Statistical analysis

Analysis was conducted using IBM SPSS Statistics for Windows, Version 25.0 (Released 2017; IBM Corp., Armonk, New York, United States), with descriptive variables expressed as mean ± standard deviation. The Student's paired t-test was utilized to compare PFT parameters between PD patients and predicted values based on age, sex, and height, and to examine differences between "off" and "on" medication states. Pearson correlation tests were used to investigate potential relationships between variables, offering a robust statistical foundation for our conclusions.

## Results

The majority of the 95 PD patients included in the study fell within the age group of 51-60 years, accounting for 47.4% of the study population, with a mean age of presentation of 55.89 ± 8.37 years. Males constituted 65.3% (n=62) of the patients, and 34.7% (n=33) were females. The mean duration of illness was 4.08 ± 3.51 years. The majority of the patients were in stage 1 of the modified Hoehn and Yahr scale at presentation; 62.1% (n=59) and 15.8% (n=15) were in stage 3. All patients exhibited bradykinesia and tremor (100%), the hallmark features of PD, while 83.2% presented with rigidity and 30.5% had postural instability. A significant portion of the patients (71.6%) were already receiving levodopa treatment at presentation, and 28.4% were drug-naive. In the assessment of pulmonary dysfunction among PD patients, the study found that 7.4% (n=7) of the patients exhibited an obstructive pattern while a predominant 73.7% (n=70) showed a restrictive pattern, and 18.9% (n=18) had normal pulmonary function. Regarding the severity of the restrictive pattern, based on FVC values, mild restriction was observed in 18.6% (n=13) of patients, moderate in 40% (n=28), and severe in 41.4% (n=29). Additionally, a weak positive correlation (r = 0.139, P = 0.021) was noted between the severity of restriction (FVC%) and the duration of PD, suggesting a mild association between disease progression and pulmonary function impairment. All these clinicodemographic details are shown in Table 1. 

**Table 1:** Clinicodemographic details of Parkinson's disease patients This table shows all the demographic and clinical details of PD patients, the various PFT abnormalities, and the correlation of severity with disease duration.

Clinicodemographic Variables	Total Patients (N=95)
Age Distribution, mean±SD	55.89 ± 8.37 years
40-50 years, n (%)	25 (26.3%)
51-60 years, n (%)	45 (47.4%)
61-70 years, n (%)	17 (17.9%)
>70 years, n (%)	8 (8.4%)
Gender Distribution	
Male, n (%)	62 (65.3%)
Female, n (%)	33 (34.7%)
Duration of Disease, mean±SD	4.08 ± 3.51 years
Modified Hoehn and Yahr Scale	
Stage 1, n (%)	59 (62.1%)
Stage 2, n (%)	7 (7.4%)
Stage 2.5, n (%)	2 (2.1%)
Stage 3, n (%)	15 (15.8%)
Stage 4, n (%)	12 (12.6%)
Clinical Features at Presentation	
Tremor, n (%)	95 (100%)
Rigidity, n (%)	79 (83.2%)
Bradykinesia, n (%)	95 (100%)
Postural Instability, n (%)	29 (30.5%)
Treatment Status at Presentation	
Previous Treatment with L-Dopa, n (%)	68 (71.6%)
No Previous Treatment with L-Dopa, n (%)	27 (28.4%)
Pattern of Pulmonary Dysfunction	
Obstructive, n (%)	7 (7.4%)
Restrictive, n (%)	70 (73.7%)
Normal, n (%)	18 (18.9%)
Severity of Restriction in Patients with Restrictive Pattern	
Mild (FVC >70% and <80%), n (%)	13 (18.6%)
Moderate (FVC 50%-69%), n (%)	28 (40%)
Severe (FVC <50%), n (%)	29 (41.4%)
Correlation between Severity of Restriction and Duration of Disease	Weak positive correlation, r = 0.139, p = 0.021

In the comparison of PFT parameters with predicted values, the observed FVC in the patients was 1.98 ± 0.87 L, significantly lower than the predicted 2.97 ± 0.58 L (P = 0.0001). The FEV1 observed in the cases also showed a decrease (1.79 ± 0.66 L) compared to the predicted (2.41 ± 0.59 L, P = 0.0001). However, the FEV1/FVC% was not significantly different, with the observed patient value at 92.94 ± 10.18% versus the predicted 79.21 ± 6.08% (P = 0.617, NS). The observed PEFR in patients was 5.07 ± 1.87 L/second, markedly lower than the predicted 7.88 ± 1.17 L/second (P = 0.0001). These comparisons between the PFT parameters of the PD patients and the predicted values are shown in Table 2.

**Table 2:** Comparison of PFT parameters observed in the study patients vs. predicted reference values p<0.05 significant PFT: pulmonary function test; PVC: forced vital capacity; FEV1: minimum forced expiratory volume in the first second; PEFR: peak expiratory flow rate

PFT parameters	Patient values, mean ± SD	Predicted reference values, mean ± SD	r value	p value
FVC (L)	1.98 ± 0.87	2.97 ± 0.58	0.549	<0.0001
FEV1 (L)	1.79 ± 0.66	2.41 ± 0.59	0.484	<0.0001
FEV1/FVC%	92.94 ± 10.18	79.21 ± 6.08	-0.052	0.617
PEFR (L/sec)	5.07 ± 1.87	7.88 ± 1.17	0.576	<0.0001

In comparing PFT parameters between the "off" and "on" stages in PD patients undergoing levodopa treatment, significant improvements were observed across all metrics. Specifically, FVC increased from 1.98 ± 0.97 L in the off stage to 2.47 ± 0.93 L in the on stage (P = 0.0001). Similarly, FEV1 rose from 1.76 ± 0.72 L to 2.13 ± 0.67 L (P = 0.0001), while the FEV1/FVC% decreased from 91.79 ± 11.28% to 87.34 ± 9.37% (P = 0.0001), indicating a return towards normalcy. PEFR also saw an increase from 4.86 ± 1.98 L/second to 5.82 ± 2.08 L/second (P = 0.0001). PD patients with both restrictive (n=49) and obstructive patterns (n=7) demonstrated notable enhancements in FVC, FEV1, FEV1/FVC ratio, and PEFR, all showing statistical significance (p < 0.05), highlighting the positive impact of levodopa treatment on pulmonary function. The PFT parameters in the off and on states of PD patients are shown in Table 3.

**Table 3:** Comparison of PFT values in on and off states in different PD patients, after levodopa administration. p<0.05 significant PFT: pulmonary function test; PVC: forced vital capacity; FEV1: minimum forced expiratory volume in the first second; PEFR: peak expiratory flow rate; PD: Parkinson's disease

Parameter	Off Stage (mean±SD)	On Stage (mean±SD)	r value	p value
Overall PD Patients				
FEV1 (L)	1.76±0.72	2.13±0.67	0.867	<0.0001
FEV1/FVC (%)	91.79±11.28	87.34±9.37	0.676	<0.0001
PEFR (L/sec)	4.86±1.98	5.82±2.08	0.944	<0.0001
PD Patients with Restrictive Pattern				
FVC (L)	1.54±0.57	2.13±0.71	0.661	<0.0001
FEV1 (L)	1.46±0.56	1.89±0.59	0.784	<0.0001
FEV1/FVC (%)	95.38±8.01	88.53±7.69	0.395	0.005
PEFR (L/sec)	4.12±1.75	5.11±1.81	0.948	<0.0001
PD Patients with Obstructive Pattern				
FVC (L)	3.72±1.18	4.15±0.89	0.948	0.001
FEV1 (L)	2.45±0.72	2.86±0.45	0.946	0.001
FEV1/FVC (%)	66.17±1.36	69.63±3.59	0.897	0.006
PEFR (L/sec)	6.96±1.61	7.54±1.54	0.962	0.001

## Discussion

Our comprehensive observational study, conducted over two years, provides significant insights into the pulmonary function abnormalities in PD patients and evaluates the therapeutic impact of levodopa. With a sample size of 95 PD patients, our research delineates crucial findings based on demographic distribution, clinical presentation, pulmonary function patterns, and the role of Levodopa. Our study predominantly identified PD patients within the 51-60 age bracket, corroborating the disease's classification as predominantly affecting middle-aged individuals. Similar observations were made from other Indian studies in the past [12,13]. This age distribution aligns with the broader epidemiological understanding of PD as a neurodegenerative disorder escalating with age. Furthermore, the male predominance observed in our cohort echoes findings from previous studies that suggest biological sex differences might influence susceptibility to PD, potentially due to differences in the nigrostriatal dopaminergic pathways and protective effects of estrogen [14-16]. We found the majority of our patients, 62.1%, were in stage 1 of the disease based on the modified Hoehn and Yahr scale staging, similar to other study reports [17]. 

On comparing the spirometric parameters between PD patients and their normal predicted reference values (age, sex, and height matched), we found significant (p<0.05) lower FVC, FEV1, and PEFR mean values in the patient population. These findings were similar to those of other studies [18,19]. The PEFR was significantly low in our study, just like in studies by Pal et al. [18] and Polatli et al. [19], suggesting poor muscular effort rather than mechanical characteristics of the lungs. On analyzing the FEV1/FVC ratio, out of a total of 95 patients, 70 patients (73.7%) had restrictive defects, and seven patients (7.4%) had obstructive defects, which was similar to earlier study results [20-22]. Contrary to the current study, many previous studies also reported an obstructive pattern in around 50% of cases of PD [23-25]. Restrictive changes occur secondary to chest wall rigidity and a reduction in lung volume secondary to kyphoscoliosis, abnormal ventilatory control, diaphragmatic dyskinesias, and pleuropulmonary complications of medications in these patients.

On correlating the duration of disease and severity of restriction, using the Pearson correlation test, we found a weak positive correlation (r = 0.139, p = 0.021), which suggests more respiratory dysfunctions as the disease advances. This is a relatively underexplored aspect of PD research and merits further investigation, considering the critical role of respiratory function in patient quality of life and mortality. This is similar to previous observations [26,27].

Our study provides compelling evidence of levodopa's positive impact on pulmonary function, with significant improvements (p<0.05) observed in FVC, FEV1, FEV1/FVC ratio, and PEFR from "off" to "on" medication states. This therapeutic effect aligns with the results of previous studies, where considerable improvement in respiratory function was seen following levodopa therapy [28-29]. The improvement in pulmonary parameters suggests levodopa's potential in alleviating respiratory dysfunction, possibly by mitigating chest rigidity and enhancing lung volumes, thereby indirectly benefiting PD patients beyond motor symptom relief. These improvements were significant (p<0.05) in both obstructive and restrictive patterns, which suggests varying mechanisms of levodopa in improving the pulmonary functions of these patients.

Limitations and recommendations

Our study is limited by a small sample size, a majority of patients in early illness stages, and the absence of longitudinal follow-up within this cohort to observe abnormalities over time. This study underscores the necessity for expanded trials involving a greater number of patients and stricter criteria. These criteria should encompass drug level monitoring, thorough respiratory testing, and evaluation of respiratory muscle strength. Subsequent studies should delve into the potential effects of optimized levodopa therapy on respiratory conditions in Parkinson's disease.

## Conclusions

This study confirms the notable presence of respiratory dysfunction in PD patients, with a higher occurrence of restrictive impairments compared to obstructive ones. It underscores the correlation between PD progression and declining respiratory function, emphasizing the necessity for timely and ongoing pulmonary assessment and treatment as part of comprehensive PD management. Additionally, it reaffirms the effectiveness of levodopa in alleviating symptoms.
